# Supplementary material for: Bis(chloroacetamidino)-Derived Heteroarene-Fused Anthraquinones Bind to and Cause Proteasomal Degradation of tNOX, Leading to c-Flip Downregulation and Apoptosis in Oral Cancer Cells
Source: Cancers (Basel). 2022 Sep 28;14(19):4719. doi: 10.3390/cancers14194719 (PMC9562014; doi:10.3390/cancers14194719)
Supplement: Supplementary file 1 [file cancers-14-04719-s001.zip › cancers-1891866-supplementary.pdf]

NMR Spectra

(A)

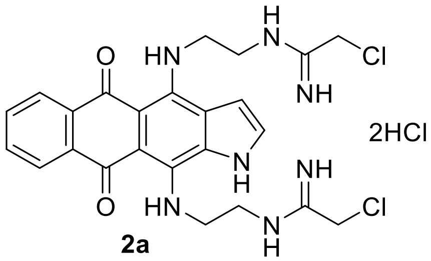

2a

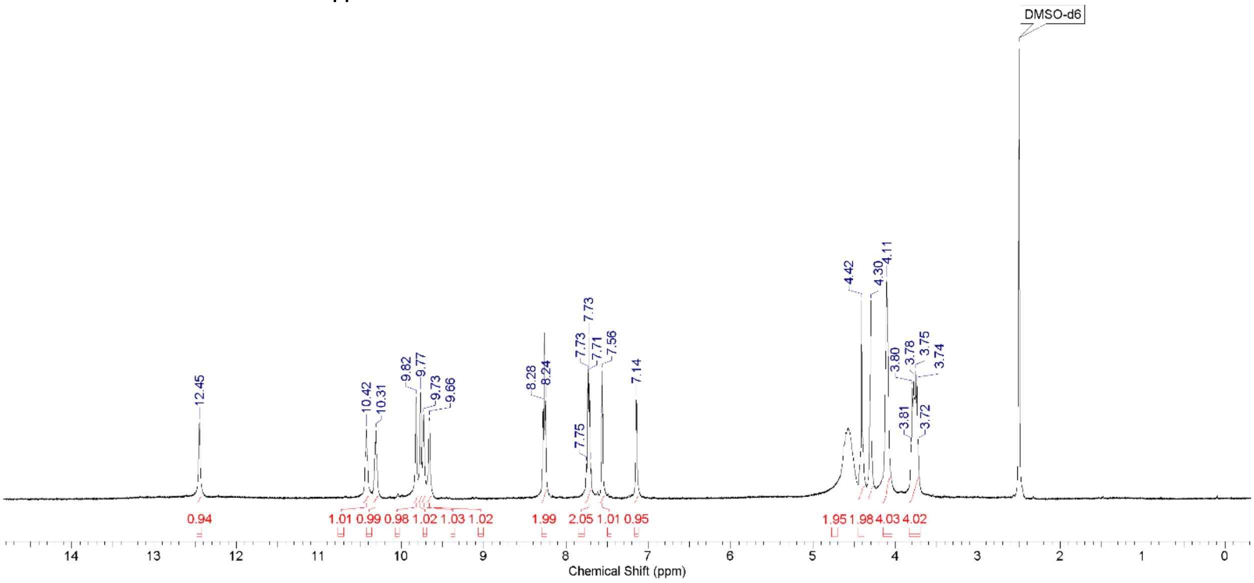

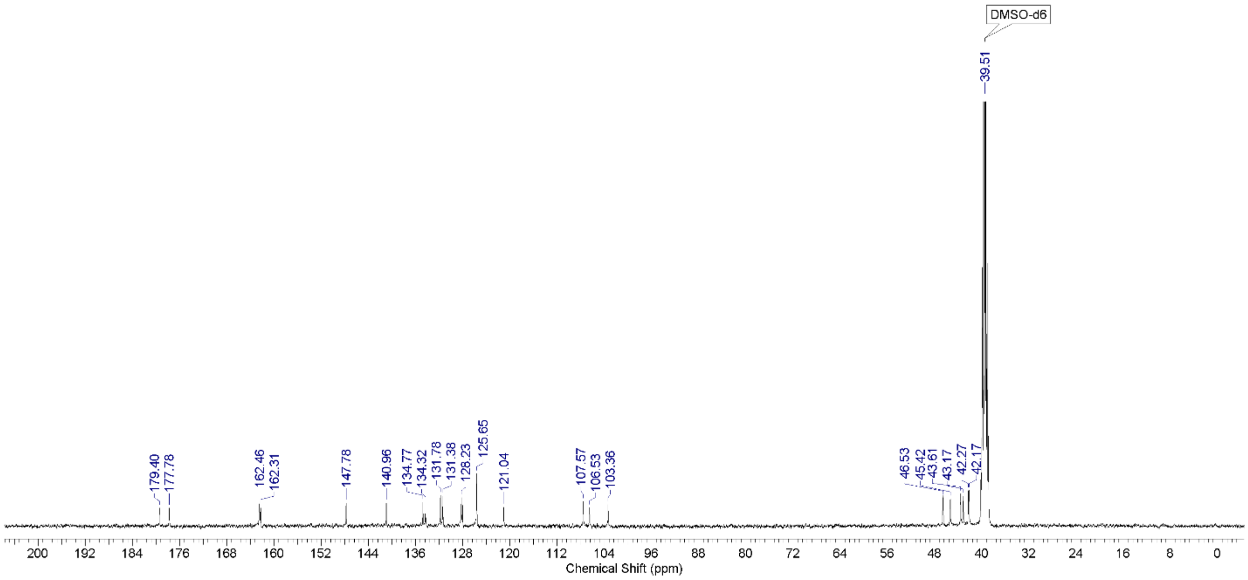

(B)

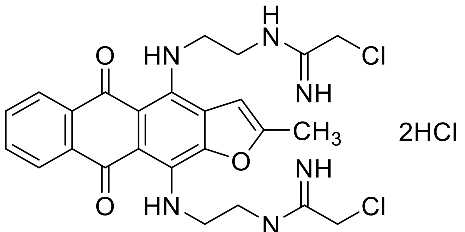
HN
H
N
Cl
NH
O
CH3
2HCl
O
NH
HN
N
H
Cl
O

2b

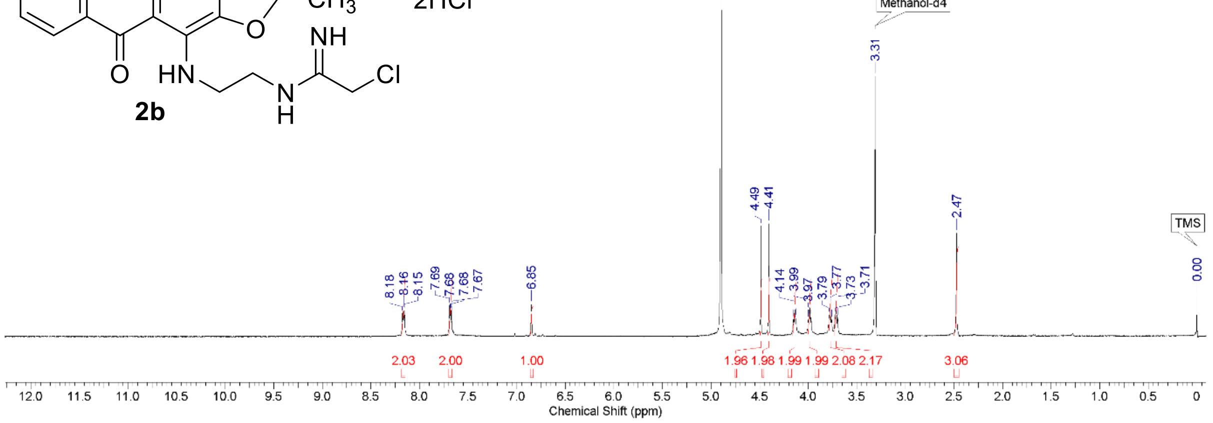
Methanol-d4
TMS
8.18 8.16 8.15 7.69 7.68 7.68 7.67 6.85
4.49 4.41 4.14 3.99 3.97 3.79 3.77 3.73 3.71 3.31 2.47 0.00
2.03 2.00 1.00 1.96 1.98 1.99 1.99 2.08 2.17 3.06
12.0 11.5 11.0 10.5 10.0 9.5 9.0 8.5 8.0 7.5 7.0 6.5 6.0 5.5 5.0 4.5 4.0 3.5 3.0 2.5 2.0 1.5 1.0 0.5 0
Chemical Shift (ppm)

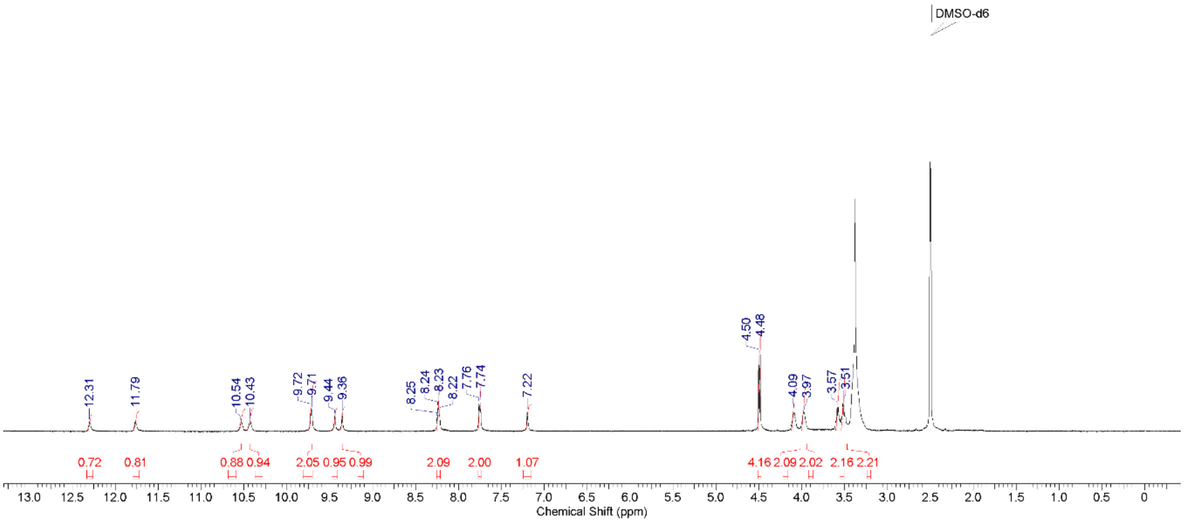
DMSO-d6
12.31 11.79 10.54 10.43 9.72 9.71 9.44 9.36 8.25 8.24 8.23 8.22 7.76 7.74 7.22
4.50 4.48 4.09 3.97 3.57 3.51
0.72 0.81 0.88 0.94 2.05 0.95 0.99 2.09 2.00 1.07 4.16 2.09 2.02 2.16 2.21
13.0 12.5 12.0 11.5 11.0 10.5 10.0 9.5 9.0 8.5 8.0 7.5 7.0 6.5 6.0 5.5 5.0 4.5 4.0 3.5 3.0 2.5 2.0 1.5 1.0 0.5 0
Chemical Shift (ppm)

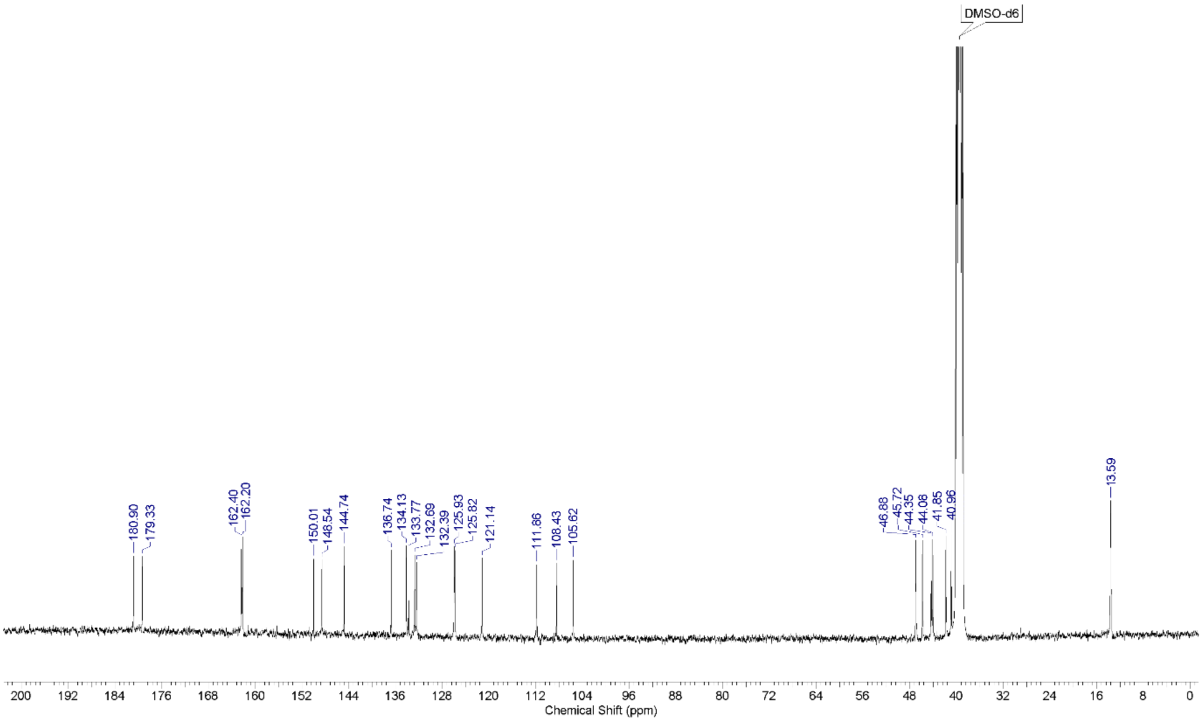
DMSO-d6
180.90 179.33 162.40 162.20 150.01 148.54 144.74 136.74 134.13 133.77 132.69 132.39 125.93 125.82 121.14 111.86 108.43 105.82
46.88 45.72 44.35 44.08 41.85 40.96 13.59
200 192 184 176 168 160 152 144 136 128 120 112 104 96 88 80 72 64 56 48 40 32 24 16 8 0
Chemical Shift (ppm)

(C)

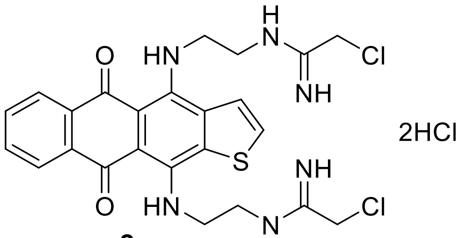
HN
H
N
Cl
NH
O
S
2HCl
HN
N
H
O

2c

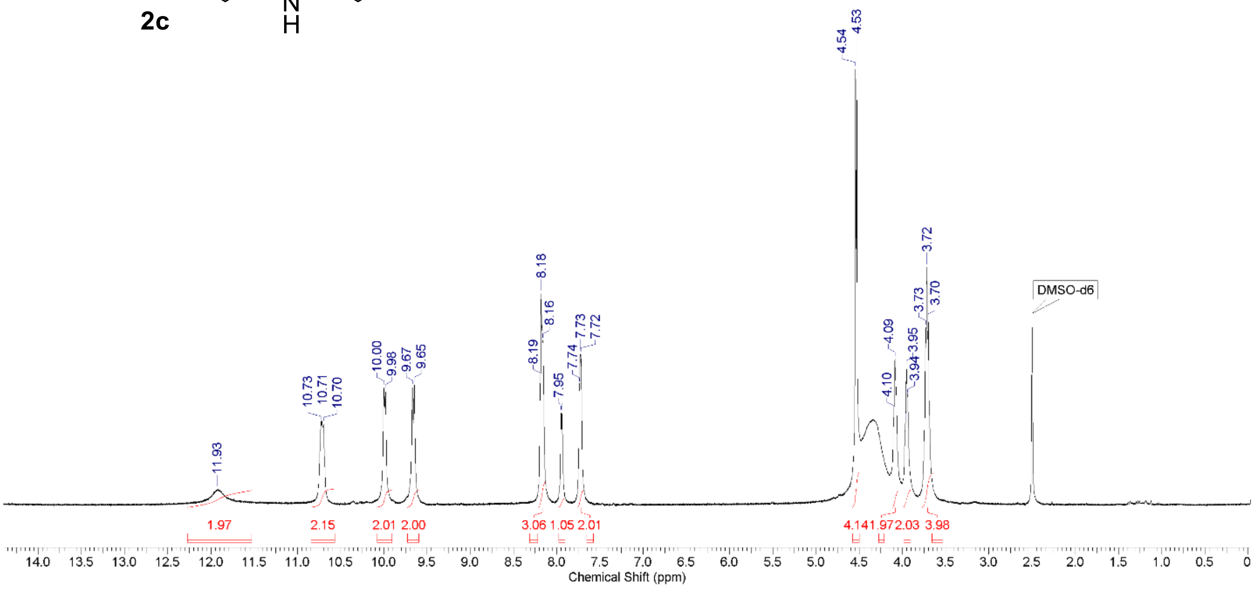
4.54
4.53
3.72
3.73
3.70
DMSO-d6
8.18
8.16
8.19
7.95
7.74
7.73
7.72
10.00
9.98
9.67
9.65
10.73
10.71
10.70
11.93
4.09
4.10
3.95
3.94
1.97
2.15
2.01
2.00
3.06
1.05
2.01
4.14
1.97
2.03
3.98
Chemical Shift (ppm)

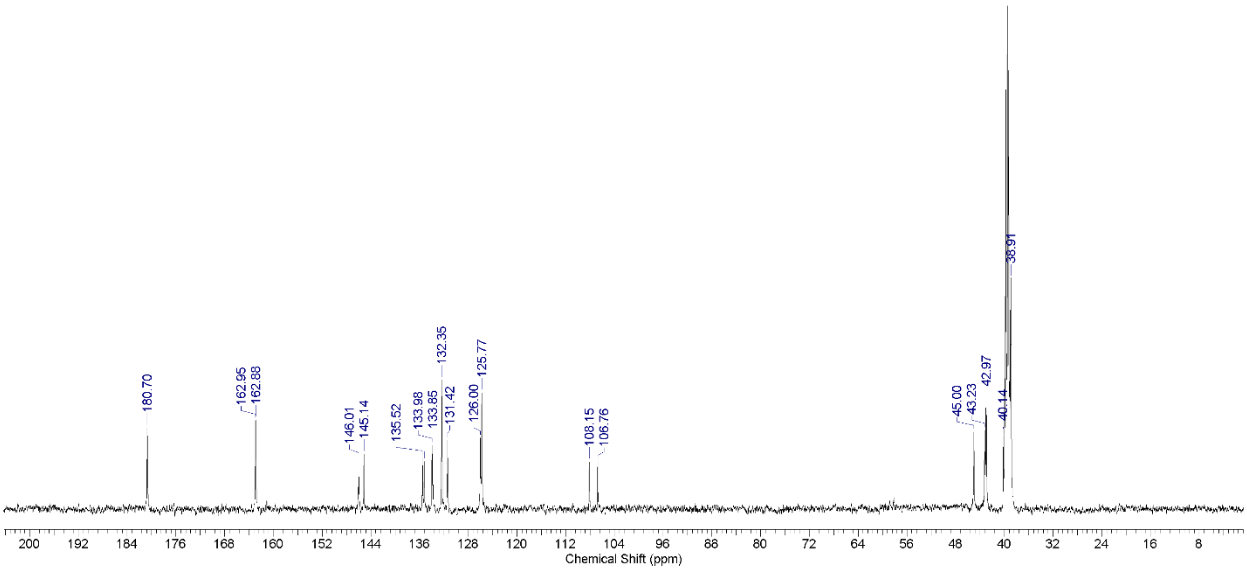
180.70
162.95
162.88
146.01
145.14
135.52
133.98
133.85
132.35
131.42
126.00
125.77
108.15
106.76
45.00
43.23
42.97
40.14
38.91
Chemical Shift (ppm)

(D)

Chromatogram
CHEK-1878-5 C:\LabSolutions\DATA\CHEK\CA F0527-004.lcd

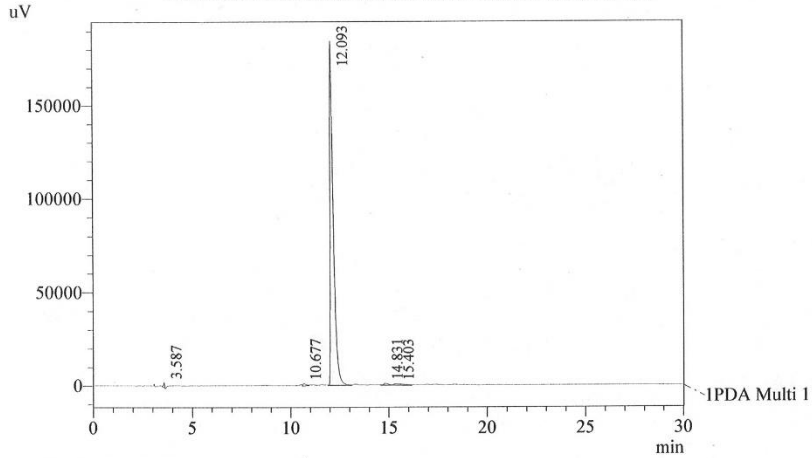

1 PDA Multi 1 / 590nm 4nm

PDA Ch1 590nm 4nm

Peak#	Ret. Time	Area	Height	Area %
1	3.587	10492	2662	0.460
2	10.677	11226	1042	0.492
3	12.093	2231145	184395	97.788
4	14.831	11409	890	0.500
5	15.403	17349	643	0.760
Total		2281622	189633	100.000

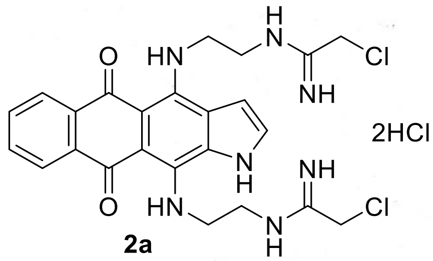

2a

Time	Unit	Command	Valu
0.01	Pumps	B.Conc	15
20.00	Pumps	B.Conc	40
30.00	Pumps	B.Conc	70
33.00	Pumps	B.Conc	15
43.00	Controller	Stop	

Shimadzu LC-20 AD; System - FOS Colon- Kromasil-100-5mkm. C-18, 4,6x250 mm. N 81348
Elution: A - H3PO4 0,01M pH 2,6; B - MeCN, fl - 1.0 ml/min, loop 20 mkl

(E)

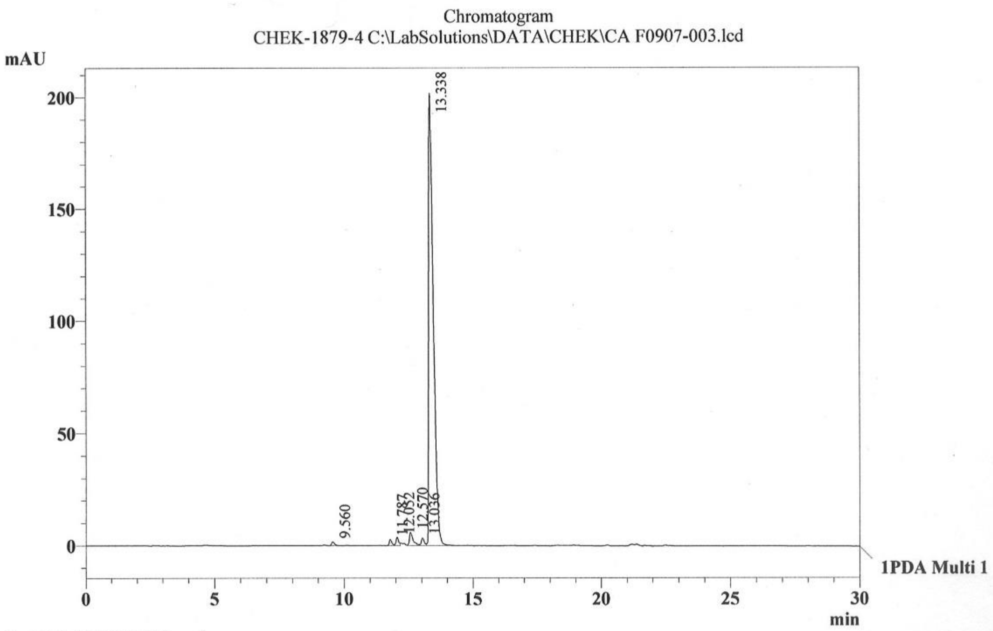

1 PDA Multi 1 / 564nm 4nm

PeakTable

PDA Ch1 564nm 4nm

Peak#	Ret. Time	Area	Height	Area %
1	9.560	12676	1600	0.458
2	11.787	19252	2778	0.696
3	12.052	24453	3614	0.884
4	12.570	58560	5914	2.116
5	13.036	24130	3293	0.785
6	13.338	2628139	201719	95.061
Total		2767210	236724	100.000

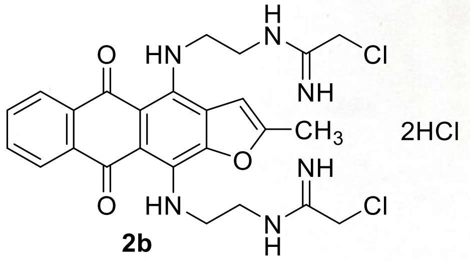

2b

Method

<<LC Program>>

Time	Unit	Command	Value
0.10	Pumps	B.Conc	15
20.00	Pumps	B.Conc	40
30.00	Pumps	B.Conc	70
33.00	Pumps	B.Conc	15
45.00	Controller	Stop	

Method Filename : FOS Av.lcm

Shimadzu LC-20AD; 2-System FOS, Colon Kromasil 100-C18,.size 5mkm, 4,6*250mm, N 62511
Elution: A - H3PO4 0.01M pH 2.6; B - MeCN, fl. 1,0 ml/min, loop 20mkl.

(F)

Chromatogram
1795 C:\LabSolutions\Data\CHEK\ST P0917-001.lcd

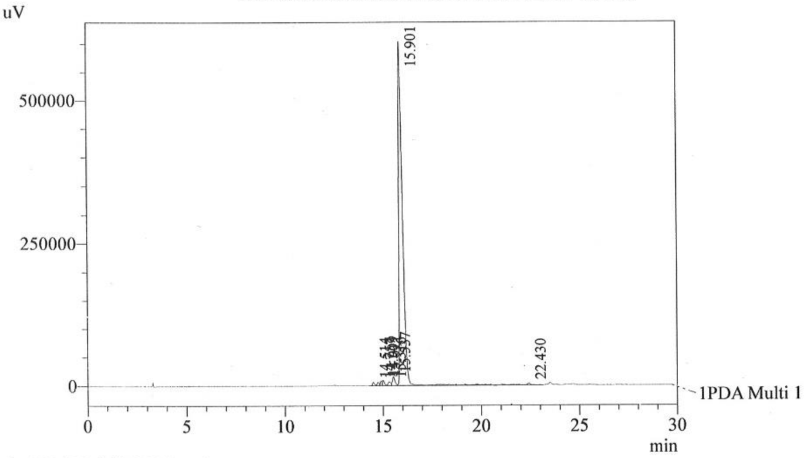

1 PDA Multi 1 / 264nm 4nm
PDA Ch1 264nm 4nm

Peak#	Ret. Time	Area	Height	Area %
1	14.514	42750	6043	0.465
2	14.757	40523	6073	0.441
3	14.909	52063	8457	0.566
4	15.011	67102	9171	0.730
5	15.316	60374	6604	0.657
6	15.537	148987	15991	1.621
7	15.901	8749029	602854	95.193
8	22.430	30972	3061	0.337
Total	19.778	9192700	692175	100.000

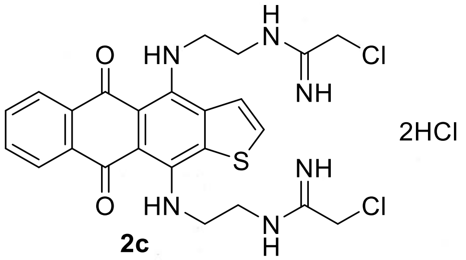

2c

Time	Unit	Command	Valu
0.01	Pumps	B.Conc	10
30.00	Pumps	B.Conc	50
33.00	Pumps	B.Conc	10
45.00	Controller	Stop	

Shimadzu LC-20 AD; System - FOS Colon- Kromasil-100-5mkm. C-18, 4,6x250 mm. N 62511
Elution: A - H3PO4 0,01M pH 2,6; B - MeCN, fl - 1.0 ml/min, loop 20 mkl

Figure S1. (A-C) NMR spectra of bis(chloroacetamidino)heteroareneanthraquinones **2a-2c**. (D-F) HPLC chromatograms of bis(chloroacetamidino)heteroareneanthraquinones 2a-2c.

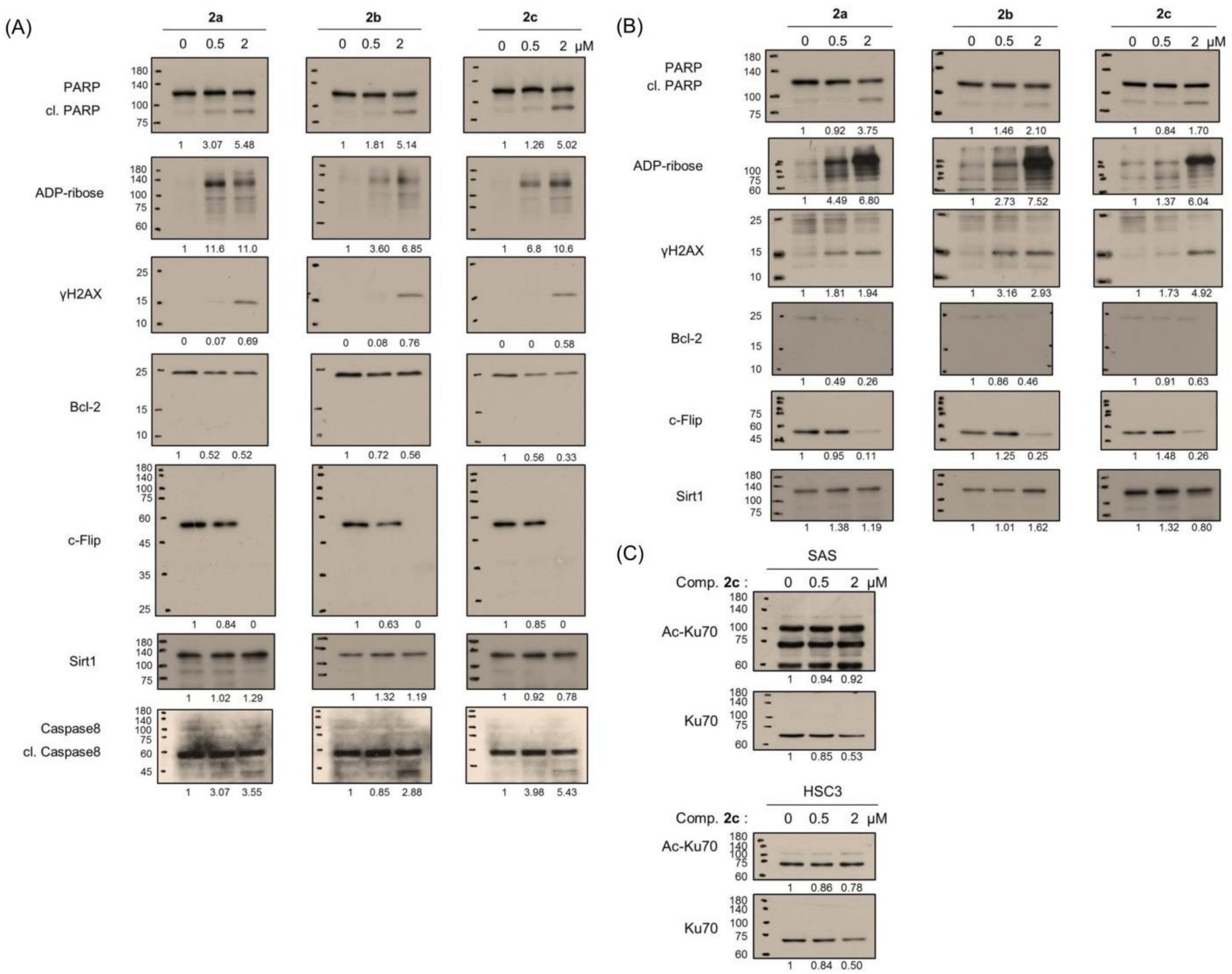

Figure S2

(A) The uncropped blots and molecular weight markers in Figure 3B for the SAS cells. (B) The uncropped blots and molecular weight markers in Figure 3B for the HSC-3 cells. (C) The uncropped blots and molecular weight markers in Figure 3C.

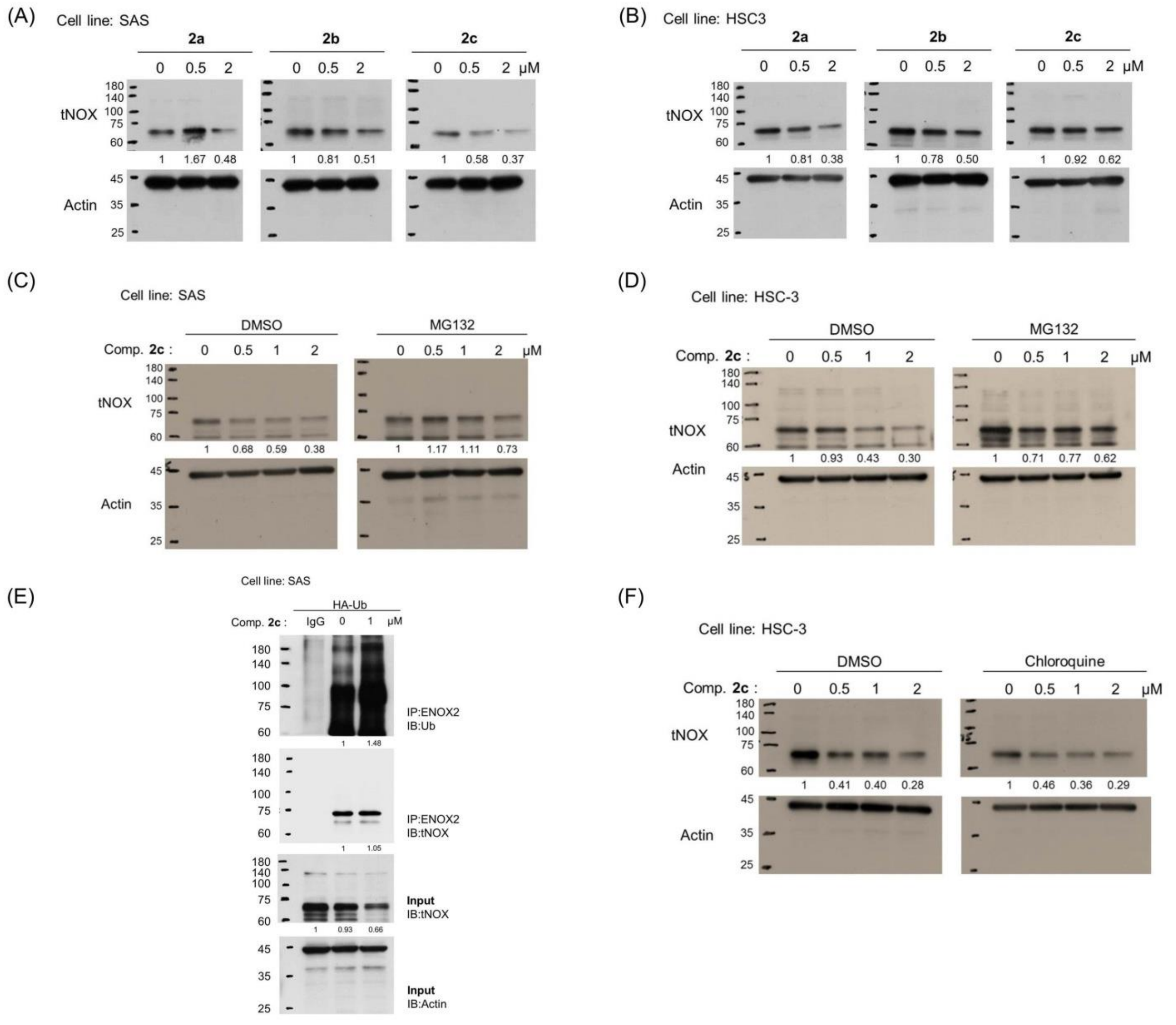

Figure S3

(A) The uncropped blots and molecular weight markers in Figure 4A for the SAS cells. (B) The uncropped blots and molecular weight markers in Figure 4A for the HSC-3 cells. (C) The uncropped blots and molecular weight markers in Figure 4B for the SAS cells. (D) The uncropped blots and molecular weight markers in Figure 4B for the HSC-3 cells. The uncropped blots and molecular weight markers in Figure 4A for the SAS cells. (E) The uncropped blots and molecular weight markers in Figure 4C for the SAS cells. (F) The uncropped blots and molecular weight markers in Figure 4D for the HSC-3 cells.

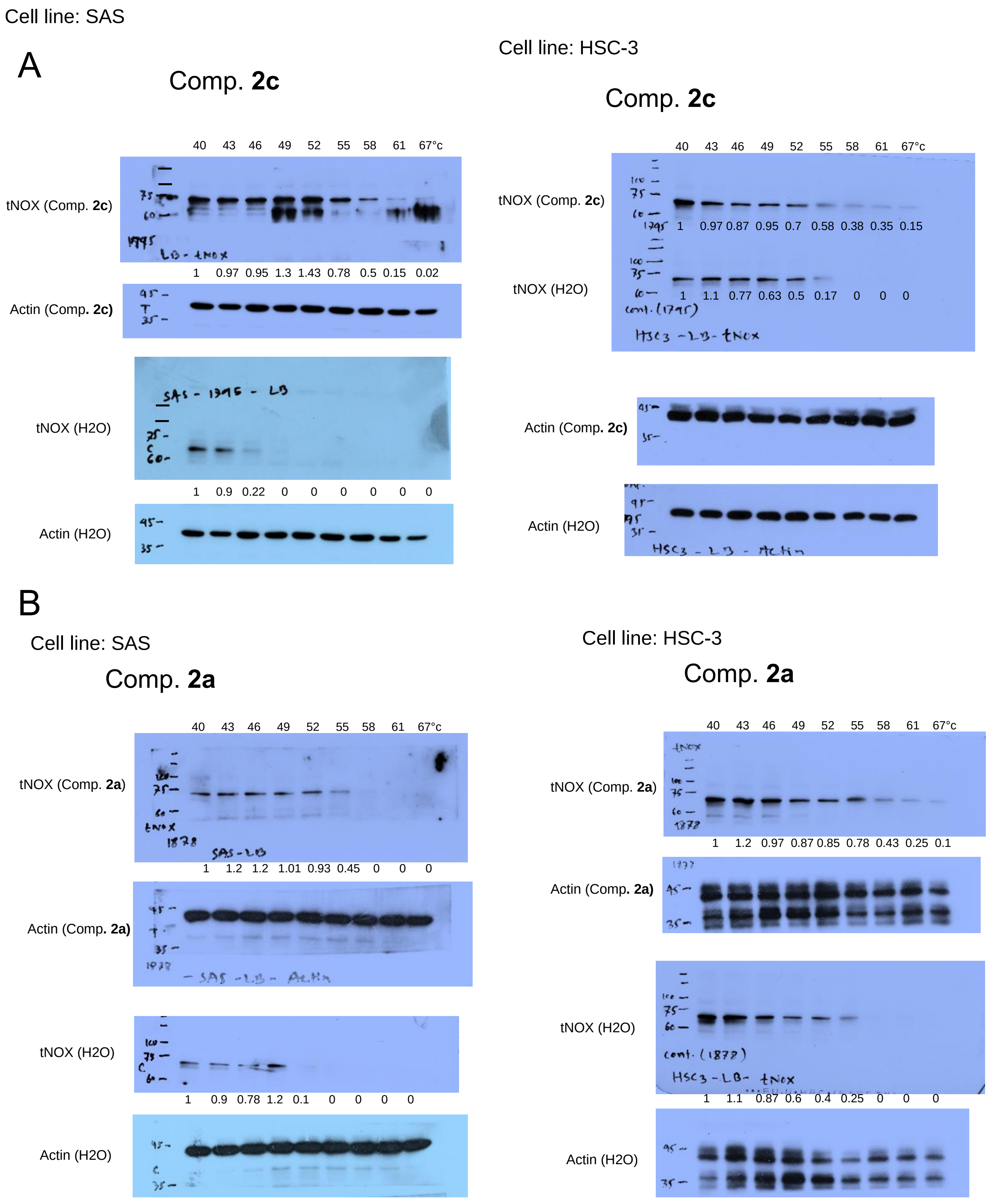
Cell line: SAS
A
Comp. 2c
40 43 46 49 52 55 58 61 67°c
tNOX (Comp. 2c)
1 0.97 0.95 1.3 1.43 0.78 0.5 0.15 0.02
Actin (Comp. 2c)
tNOX (H2O)
1 0.9 0.22 0 0 0 0 0 0
Actin (H2O)
Cell line: HSC-3
Comp. 2c
40 43 46 49 52 55 58 61 67°c
tNOX (Comp. 2c)
1 0.97 0.87 0.95 0.7 0.58 0.38 0.35 0.15
tNOX (H2O)
1 1.1 0.77 0.63 0.5 0.17 0 0 0
Actin (Comp. 2c)
Actin (H2O)
B
Cell line: SAS
Comp. 2a
40 43 46 49 52 55 58 61 67°c
tNOX (Comp. 2a)
1 1.2 1.2 1.01 0.93 0.45 0 0 0
Actin (Comp. 2a)
tNOX (H2O)
1 0.9 0.78 1.2 0.1 0 0 0 0
Actin (H2O)
Cell line: HSC-3
Comp. 2a
40 43 46 49 52 55 58 61 67°c
tNOX (Comp. 2a)
1 1.2 0.97 0.87 0.85 0.78 0.43 0.25 0.1
Actin (Comp. 2a)
tNOX (H2O)
1 1.1 0.87 0.6 0.4 0.25 0 0 0
Actin (H2O)

C

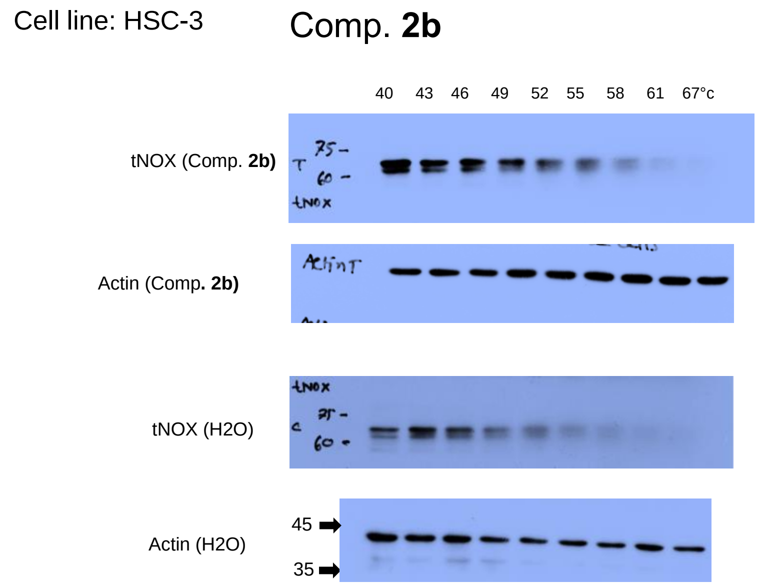

Figure S4

(A) The uncropped blots and molecular weight markers in Figure 5A. (B) The uncropped blots and molecular weight markers in Figure 5B. (C) The uncropped blots and molecular weight markers in Figure 5C.
